# Polymorphism rs7555523 in *transmembrane and coiled-coil domain 1* (*TMCO1*) is not a risk factor for primary open angle glaucoma in a Saudi cohort

**DOI:** 10.1186/s12952-016-0060-1

**Published:** 2016-09-29

**Authors:** Altaf A. Kondkar, Ahmed Mousa, Taif A. Azad, Tahira Sultan, Abdullah Alawad, Saleh Altuwaijri, Saleh A. Al-Obeidan, Khaled K. Abu-Amero

**Affiliations:** 1 Ophthalmic Genetics Laboratory, Department of Ophthalmology, College of Medicine, King Saud University, P.O. Box 245, Riyadh, 11411 Saudi Arabia; 2 Glaucoma Research Chair, Department of Ophthalmology, College of Medicine, King Saud University, Riyadh, Saudi Arabia; 3 National Center for Stem Cell Technology (NCSCT), Life Sciences and Environmental Research Institute, King Abdulaziz City for Science and Technology, Riyadh, Saudi Arabia; 4 SAAD Research & amp; Development Center, Clinical Research Lab., SAAD Specialist Hospital, Al Khobar, Saudi Arabia; 5 Qassim University, Burayadh, Saudi Arabia; 6 Department of Ophthalmology, College of Medicine, Jacksonville, FL USA

**Keywords:** POAG, rs7555523, Saudi Arabia, Transmembrane and coiled-coil domain 1

## Abstract

**Background:**

We investigated whether polymorphism rs7555523 (A > C) in human *transmembrane and coiled-coil domain 1* (*TMCO1*) gene is a risk factor for primary open angle glaucoma (POAG) in a Saudi cohort.

**Methods:**

A cohort of 87 unrelated POAG cases and 94 control subjects from Saudi Arabia were genotyped using Taq-Man® assay. The association of genotypes with POAG and other glaucoma specific clinical indices was investigated.

**Results:**

The genotype and allele frequency of polymorphism rs7555523 at *TMCO1* did not show any statistically significant association with POAG as compared to controls. The minor allele frequency was 0.103 in cases and 0.085 in controls. Except for awareness of glaucoma (*p* = 0.036), no significant association of genotypes were seen with glaucoma specific clinical indices such as intraocular pressure (IOP), cup/disc ratio and number of anti-glaucoma medications used. Binary logistic regression analysis (adjusted for age and gender) showed that age was a significant indicator for the development of glaucoma in this group (adjusted odds ratio = 1.2; 95 % confidence interval = 1.078–1.157; *p* < 0.001).

**Conclusion:**

Our study was unable to replicate the findings of previously reported association for polymorphism rs7555523 in *TMCO1* with POAG and related clinical indices such as IOP and cup/disc ratio indicating that this variant is not a risk factor for POAG in the Saudi cohort.

## Background

Glaucoma, a neurodegenerative disease is characterized by progressive damage of retinal ganglion cells (RGCs) resulting in characteristic cupping of the optic nerve head and loss of peripheral vision [1]. Primary open angle glaucoma (POAG) is the second most common form of glaucoma in Saudi Arabia which is clinically characterized by an open and normal anterior iridocorneal chamber angle [2]. Aging, gender, African ancestry, family history, elevated intraocular pressure (IOP), central corneal thickness, and myopia are some of the well-recognized risk factors associated with POAG pathogenesis [3]. Although POAG is clinically well-defined, the biological basis of the disease is not well understood and factors contributing to its progression are not fully characterized.

Genetic studies represent an important tool to identify genes and molecular pathways involved in disease pathogenesis. POAG is genetically complex with largely polygenic and multifactorial inheritance [4]. Using a genome-wide and candidate gene approach, population-based genetic studies have identified several genes and genetic variants associated with POAG and related quantitative endophenotype traits [5]. A genome-wide association study (GWAS) in Australians of European descent identified a susceptibility locus at *transmembrane and coiled-coil domains 1* (*TMCO1*) [6]. van Koolwijk et al. performed a GWAS for IOP in POAG patients of European descent and identified single nucleotide polymorphism (SNP) 7555523, located in *TMCO1* suggesting a role in IOP regulation [7]. *TMCO1* gene is located 7.6 MB upstream of the known POAG gene, *myocilin C* (*MYOC)*, on chromosome 1q24.1. It encodes a transmembrane protein with a coiled-coil domain that may localize to the Golgi apparatus and endoplasmic reticulum or to the mitochondria in different cell types with a plausible role in apoptosis of RGCs [6, 8]. TMCO1 is highly expressed in the human ciliary body, trabecular meshwork and retina [7, 9, 10]. However, precise role of *TMCO1* in POAG pathogenesis is still unclear.

So far, there are no published reports of association studies at the *TMCO1* locus in the Middle East population. In the current study, we investigated the association of *TMCO1* SNP rs7555523 with POAG in a Saudi cohort.

## Methods

### Study design and setting

This case–control genetic association study was conducted between November 2015 through February 2016 at King Abdulaziz University Hospital, King Saud University, Riyadh, Saudi Arabia.

### Study population

We recruited 87 Saudi adult-POAG patients who satisfied strict the following clinical criteria: i) appearance of the disc or retinal nerve fiber layer e.g., thinning or notching of disc rim, progressive changes, nerve fiber layer defect; ii) presence of characteristic abnormalities in visual field (e.g., arcuate scotoma, nasal step, paracentral scotoma, generalized depression) in the absence of other causes or explanation; iii) age >40 years, and iv) open anterior chamber angles bilaterally on gonioscopy. Exclusion criteria included evidence of secondary glaucoma, e.g., pigmentary dispersion syndrome, pseudoexfoliation, history of steroid use, or ocular trauma. All cases had onset of glaucoma after age 40 (adult onset POAG). Patients were recruited from the glaucoma clinic at King Abdulaziz University Hopsital after signing an informed consent approved by the institutional review board (proposal number # 08–657). A second group healthy controls (*n* = 94) of Saudi origin and free from glaucoma by examination were recruited. Inclusion criteria included: >40, normal IOP, open angles on gonioscopy and normal optic nerves upon examination.

### Genotyping

Genotyping of intronic polymorphism, rs7555523 (g.165718979A > C), of the *TMCO1* gene (NC_000001.10) was performed using the TaqMan® SNP Genotyping assay ID: C_29621671_10 (Applied Biosystems Inc., Foster City, CA, USA) on ABI 7500 real-time PCR system (Applied Biosystems). Each PCR reaction was performed in a 96-well plate in a total volume of 25 μL consisting of 1X TaqMan® Genotyping Master Mix (Applied Biosystems), 1X SNP Genotyping Assay Mix, 20 ng DNA, and two no template (negative) controls under cycling conditions recommended by the manufacturer [11]. Genotypes of *TMCO1 rs7555523* SNP were identified using the automated 2-color allele discrimination software on ABI 7500 on a two-dimensional graph.

### Statistical analysis

The continuous variables were presented as mean (± Standard Deviation, SD) and tested by Student’s *t*-test. Categorical variables were presented as frequencies and percentages. Hardy-Weinberg Equilibrium (HWE) deviation was tested by Pearson’s Chi^2^ test. Odds ratio (OR) was calculated and Chi^2^ test was used to detect any association between different characteristics and the genetic profiles (Fisher Exact test when applicable). Mann–Whitney U test was used to investigate whether there was any significant difference between the genotypes and clinical variables. The confidence interval (CI) level was set to 95 % and a *p* value below 0.05 was considered statistically significant. Data were analyzed using SPSS® version 22.0 (IBM Inc., Chicago, Illinois, USA).

## Results

In the current study we recruted 87 adult-POAG patinets with confirmed diagnosis as POAG and a matching group of 94 subjects that served as controls after a confirmed clinical examination that identified them as “glaucoma free”, see methods.

As shown in Table 1 cases had a mean age of 61.1 years (ranging from 43 to 74 years) where 52 (59.8 %) of them were male and 35 (40.2 %) were female. On the other hand, controls showed a mean age of 56.5 years (range 45–70 years) where 69 (73.4 %) of them were male and 25 (26.6 %) were female. Despite the fact that males were more than females in both study groups, this diffrence was not statastcalliy significant. No significant difference was observed between cases and controls in terms of all demographic, systemic co-morbidity and glaucoma specific indices except for the family history of glaucoma and awareness of having glaucoma (*p* = 0.006 and <0.0001, respectively).

**Table 1:** Demographic and clinical characteristics of cases and control

Variables	Category	Cases (*N* = 87) No. (%)	Controls (*N* = 94) No. (%)	*p* value
Demographic Characteristics				
Age in years, Mean (±SD)		61.1 (±12.6)	56.5 (±10.4)	0.124
Gender	Male	52 (59.8)	69 (73.4)	0.052
	Female	35 (40.2)	25 (26.6)	
Systemic Diseases				
Diabetes mellitus	Present	43 (49.4)	37 (39.4)	0.173
Hypertension	Present	42 (48.3)	36 (38.3)	0.176
Coronary artery disease	Present	5 (5.7)	2 (2.1)	0.264
Hypercholesterolemia	Present	13 (14.9)	7 (7.4)	0.108
Health Awareness/Behavior				
Family history of glaucoma	Yes	11 (12.6)	2 (2.1)	0.006
Smoking	Yes	36 (41.4)	29 (30.9)	0.142
Awareness to glaucoma	Yes	14 (16.1)	0.0 (0.0)	<0.0001

The genotype frequencies in both the cases and control groups did not deviate significantly from the HWE (*p* > 0.05). The wildtype genotype (A/A) was predominant in both cases and controls (*n* = 69 (79.3 %) and *n* = 78 (83 %), respectively) with a slightly increased frequency of heterozygous (C/A) genotype in cases (18; 20.7 %) versus controls (16; 17 %). No homozygous mutant genotype (C/C) was observed in both the groups. The cases were 1.3 times more likely to encounter a variation, however, the distribution was non-significant (OR = 1.3; 95 % CI = 0.563–2.891; *p* = 0.571). Likewise, the same effect was detected while comparing the frequency of the wildtype “A” allele to the mutated “C” allele, with a similar OR (1.3) and a non-significant p value (0.591). The genotype and allele frequency distribution is shown in Table 2.

**Table 2:** Comparison of genotype and allele distribution of SNP rs7555523 in cases and controls

Genotype	Cases (*N* = 87) No. (%)	Controls (*N* = 94) No. (%)	Odds Ratio	95 % Confidence Interval	*p* value
A/A	69 (79.3)	78 (83)	-	-	-
A/C	18 (20.7)	16 (17)	1.3	[0.563–2.891]	0.571
Allele					
A	156 (89.7)	173 (91.5)	-	-	-
C	18 (10.3)	16 (8.5)	1.3	[0.578–2.713]	0.591

Furthermore, we evaluated the effect of genotype on demographic, systemic diseases and glaucoma specific indices among POAG cases as demonstrated in Table 3. Although there was a preponderance of male subjects in both the genotype groups and the subjects with A/C genotypes were found to be slighter younger but there was no statistically significant difference in terms of age (*p* = 0.644) and gender (*p* = 0.421). Similarly, except for awareness of having glaucoma variable (*p* = 0.036), no statistically significant difference was observed in terms of systemic diseases and health awareness/ behavior characteristics. However, more importantly, none of the glaucoma specific indices such as IOP, cup/disc ratio and number of anti-glaucoma medications showed any statistically significant difference between the two genotype groups.

**Table 3:** Effect of genotypes on demographic and other clinical characteristics among POAG cases

Variables	Category	Genotypes		*p* value
		A/A (*N* = 69) No. (%)	A/C (*N* = 18) No. (%)	
Demographic Characteristics				
Age in years, Mean (±SD)	-	61.5 (13.2)	59.8 (9.9)	0.644
Gender	Male	43 (62.3)	9 (50.0)	0.4212
	Female	26 (37.7)	9 (50.0)	-
Systemic Diseases				
Diabetes mellitus	Present	37 (53.6)	6 (33.3)	0.205
Hypertension	Present	34 (49.3)	8 (44.4)	0.920
Coronary artery disease	Present	4 (5.8)	1 (5.6)	0.971
Hypercholesterolemia	Present	9 (13.0)	4 (22.2)	0.357
Health Awareness / Behavior				
Family history of glaucoma	Yes	9 (13)	2 (11.1)	0.988
Smoking	Yes	26 (38.2)	10 (55.6)	0.272
Awareness to glaucoma	Yes	14 (20.3)	0.0 (0.0)	0.036
Glaucoma Specific Indices				
Intraocular pressure in mmHg, Mean (±SD)	-	35.0 (7.5)	34.8 (8.0)	0.778
Cup/disc ratio, Mean (±SD)	-	0.72 (0.18)	0.7 (0.2)	0.740
Number of anti-glaucoma medications, Mean (±SD)	-	2.9 (0.7)	2.8 (0.6)	0.765

In addition, to investigate the effect of harboring a mutated genotype on having glaucoma, we performed a binary logistic regression analysis (adjusted for age and gender). The analysis showed that patients with mutated genotype seems to be 1.3 times more likely to get the disease (POAG), however, the OR was not found to be statistically significant (adjusted OR = 1.3; 95 % CI = 0.534–3.261; *p* = 0.548). Nevertheless, adjustment of age and gender revealed that although non-significant, females were 1.6 times more likely to get glaucoma than males (adjusted OR = 1.6; 95 % CI = 0.778–3.428; *p* = 0.195); and not surprisingly, age was found to be a significantly strong indicator for the development of glaucoma in this group (adjusted OR = 1.2; 95 % confidence interval = 1.078–1.157; *p* < 0.001).

## Discussion

Given the complexity and genetic mutational heterogeneity of POAG recent GWASs have identified a number of polymorphisms in multiple loci/genes including *caveolin* (*CAV1/CAV2*) [12], *atonal homolog 7* (*ATOH7*) [13], *sin oculis homeobox* (*SIX1/SIX6*) [14], *cyclin-dependent kinase inhibitor 2B antisense RNA 1* (*CDKN2B-AS1*) [6, 14] and *TMCO1* [6, 14] that may contribute to the development and/or progression of POAG in various ethnic groups. In this study, we investigated whether SNP rs7555523 (A > C) in *TMCO1* gene is a risk factor for POAG in a Saudi cohort.

Genetic variation in *TMCO1* has been associated with POAG [6, 15]. The association of the *TMCO1* locus with POAG has been replicated in another GWAS [7]. Similarly, *TMCO1* loci (including rs7555523) showed significant association with POAG and high-tension glaucoma in a Han Chinese population [16]. However, conflicting results have been observed for populations of African ancestry [17, 18]. The frequency distribution of polymorphism(s) is known to vary significantly across the different ethnic groups. SNP rs7555523 has been reported to have a minor allele frequency (MAF) of 0.12 in Caucasians [7], 0.016 in Han Chinese [16] and 0.132 in Sub-Saharan African [http://www.ncbi.nlm.nih.gov/projects/SNP/snp_ref.cgi?rs=7555523]. The MAF observed in our POAG Saudi cohort was 0.103 which is slightly lower than the Caucasians and Africans, but much higher than the Han Chinese (Asians). However, in contrast to the Caucasian and Chinese studies, the genotype and allele frequency of rs7555523 were not found to be an independent risk factor of POAG in our cohort indicating that this SNP may not have a significant role in Saudi POAG as compared to Caucasians and Chinese.

Family history, aging, cigarette smoking, diabetes and hypertension are well recognized risk factors of POAG [19, 20]. Sharma et al. has shown that POAG patients carrying the risk allele of SNP rs4656461 near the *TMCO1* gene tend to have an earlier age at diagnosis of glaucoma [10]. However, in our study, although the subjects with mutant heterozygous (A/C) genotypes were found to be slighter younger but the difference was statistically non-significant. Likewise, SNP rs7555523 is located in a region which has been previously suggested to be linked with blood pressure [21]. In our study, however, none of the systemic diseases including hypertension were found to be significantly associated with this genotype.

*TMCO1* is a highly evolutionary conserved gene of largely unknown function [8, 22]. A homozygous frameshift mutation in *TMCO1* has been associated with a rare recessive syndrome known as “TMCO1 defect syndrome” consisting of craniofacial dysmorphism, skeletal anomalies and mental retardation [22]. It is still unclear how this gene contributes to the pathogenesis of glaucoma. Studies suggest that *TMCO1* may contribute to POAG through the pathway of IOP elevation [7, 16]. However, we did not find any significant association between heterozygous (A/C) genotype and clinical indices important for glaucoma such as IOP, cup-to-disc ratio and number of anti-glaucoma medications. It is noteworthy to mention here that *TMCO1* interacts with another known POAG susceptibility gene, *CAV1* via the von Hippel-Lindau (VHL) tumor suppressor protein-containing E3 ubiquitin ligase [7, 23]; and we have previously shown that SNP rs4236601 in *CAV1*/*CAV2* is not a risk factor for POAG in Saudi population [24], plausibly suggesting that *TMCO1* may not have an important role in POAG pathogenesis in this population. However, considering the small sample size in this study and the fact that there was no homozygous mutant (C/C) genotype observed in our sample population these observations may require further validation in a large sample population.

## Conclusion

Our study was unable to replicate the findings of previous association reported for variant rs7555523 in *TMCO1* with POAG and important clinical indices such as IOP and cup/disc ratio indicating that this SNP is not a risk factor for POAG or its important clinical indices in the Saudi cohort.

## Abbreviations

ATOH7: Atonal homolog 7
CAV1/CAV2: Caveolin
CDKN2B-AS1: Cyclin-dependent kinase inhibitor 2B antisense RNA 1
GWAS: Genome-wide association study
HWE: Hardy-Weinberg Equilibrium
IOP: Intraocular pressure
MAF: Minor allele frequency
MYOC: Myocilin C
OR: Odds ratio
POAG: Primary open angle glaucoma
RGCs: Retinal ganglion cells
*SIX1/SIX6*: *Sin oculis homeobox*
SNP: Single nucleotide polymorphism
*TMCO1*: *Transmembrane and coiled-coil domain 1*
VHL: Von Hippel-Lindau
